# Polymeric nanocapsules embedded with ultra-small silver nanoclusters for synergistic pharmacology and improved oral delivery of Docetaxel

**DOI:** 10.1038/s41598-018-30749-3

**Published:** 2018-09-06

**Authors:** Muhammad Farhan Sohail, Syed Zajif Hussain, Hamid Saeed, Ibrahim Javed, Hafiz Shoaib Sarwar, Akhtar Nadhman, Zil-e- Huma, Mubashar Rehman, Sarwat Jahan, Irshad Hussain, Gul Shahnaz

**Affiliations:** 1 Department of Pharmacy, Faculty of Biological Sciences, Quaid-i-Azam University, Islamabad, 45320 Pakistan; 2 Department of Chemistry and Chemical Engineering, SBA School of Science and Engineering (SBASSE), Lahore University of Management Sciences (LUMS), Lahore, 54792 Pakistan; 3 Riphah Institute of Pharmaceutical Sciences, Riphah International University, Lahore Campus, Lahore, Pakistan; 4 University College of Pharmacy, University of the Punjab, Allama Iqbal Campus, Lahore, Pakistan; 5 ARC Centre of Excellence in Convergent Bio-Nano Science and Technology, Monash Institute of Pharmaceutical Sciences, Monash University, 381 Royal Parade, Parkville, VIC, 3052 Australia; 6 Department of Pathology, Ohio State University Medical Center, Columbus, OH USA; 7 Institute of Integrative Biosciences, CECOS University, Phase VI, Hayatabad, Peshawar, Pakistan; 8 Department of Pharmacy, University of Lahore – Gujrat Campus, Gujrat, 50700 Pakistan; 9 US-Pakistan Center for Advanced Studies in Energy (USPCAS-E), University of Engineering & Technology (UET), Peshawar, Pakistan; 10 Department of Animal Sciences, Faculty of Biological Sciences, Quaid-i-Azam University, Islamabad, 45320 Pakistan

## Abstract

Despite of the remarkable cytotoxic and imaging potential of ultra-small metal nanoclusters, their toxicity-free and targeted delivery to cancerous cells remains a substantial challenge that hinders their clinical applications. In this study, a polymeric scaffold was first synthesized by grafting folic acid and thiol groups to chitosan (CS) for cancer cell targeting and improved gastric permeation. Furthermore, silver nanocluster (Ag NCs) were synthesized *in situ*, within CS scaffold by microwave irradiation and core-shell nanocapsules (NCPs) were prepared with hydrophobic docetaxel (DTX) in the core and Ag NCs embedded CS in the shell. A significant cytotoxicity synergism (~300 folds) was observed for DTX with co-delivery of Ag NCs against breast cancer MDA-MB-231 cells. Following oral administration, the DTX-Ag-NCPs increased bioavailability due to enhanced drug transport across gut (9 times), circulation half-life (~6.8 times) and mean residence time (~6.7 times), as compared to the control DTX suspension. Moreover, 14 days acute oral toxicity of the DTX-Ag-NCPs was performed in mice and evaluated for changes in blood biochemistry parameters, organ to body weight index and histopathology of liver and kidney tissues that revealed no significant evidence of toxicity suggesting the safety and efficiency of the DTX-Ag-NCPs as hybrid nanocarrier for biocompatible delivery of metal nanoclusters.

## Introduction

Nanotechnology is an emerging field in medical sciences enabling the use of nanoscale metallic, polymeric materials or their composites for various biomedical applications. Nanoparticles exhibit inimitable properties depending upon their size, morphology and surface chemistry compared to their parent bulk matter^1^. Metal based drug nanocarriers have evolved as efficient therapeutic moieties with diverse potentials. For this purpose, metal nanoparticles including gold (Au), silver (Ag), iron oxide (Fe) and zinc oxide (ZnO) have shown tremendous potential in therapeutics (anti-bacterial, anti-fungal and anti-cancer) and diagnostics (MRI, PET, SPECT, Fluorescent NCs, quantum dots) application^2–6^. Among these nanomaterials, nanoparticles and complexes of Ag are known for having antimicrobial and cytotoxic effects since decades^7^. Moreover, because of high nonspecific cytotoxicity and lack of delivery strategies, Ag nanoparticles could not be used therapeutically for treating cancer^8^. Clinical applications of the metallic nanoclusters will remain limited unless the challenge of toxicity is overcome^9^.

Biodegradable polymers like chitosan (CS) and its derivatives (thiolated CS, pre-activated CS etc.) have proven their potential in drug delivery, as capping agents for the synthesis of stable metal nanoclusters and other biomedical applications due to their high degree of biodegradation and biocompatibility^10,11^. Thiolated CS has shown superior potential in oral drug delivery providing advantages like mucoadhesion, P-gp inhibition, paracellular transportation through enterocytes and evasion from plasma proteome association which can mask the intended biological interaction of nanostructures and clear them from the bio-circulation^10,12,13^. Polymeric nanomaterials also provide advantages when interacting with blood cells, in contrast to surfactants based micellar nano-systems^14,15^. Docetaxel (DTX) is a clinically proven drug against numerous cancers including breast neoplastic malignancy^16,17^. Also, it shows low oral bioavailability due to extreme hydrophobicity, P-glycroprotein (P-gp) mediated efflux and first pass effect^18^. However, commercially available DTX (Taxotere®) is associated with hypersensitive reactions because of polysorbate and alcohol required for its solubilization leading to infusion related side effects and high cost. Therefore, nanoparticles based oral delivery emerged as an appealing strategy to be explored for improved oral bioavailability of DTX. Oral delivery of DTX results in improved patient compliance due to lack of I.V. injection hazards, thus, enhanced quality of life and economic aspects^19–21^.

**Figure 1:**
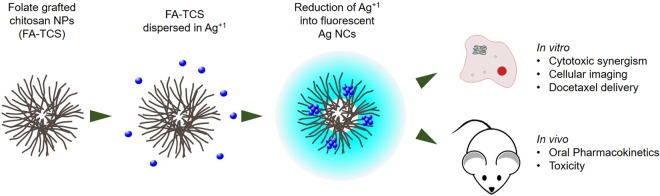
Synthesis of fluorescent AgNCs containing chitosan NPs. The Ag ions were reduced *via* UV irradiation into ultra-small nanoclusters (NCs) and explored for *in vitro* and *in vivo* drug delivery and toxicology of docetaxel.

Herein, we report DTX loaded hybrid nanocapsules (DTX-Ag-NCPs) of folate grafted-thiolated CS with embedded sliver nanocluster for synergistic cytotoxicity. The synthesized DTX-Ag-NCPs were characterized for their physicochemical, optical and biological parameters. Moreover, these hybrid nanocapsules were evaluated for acute oral toxicity to establish a broader picture of the DTX-Ag-NCPs as drug carrier (Fig. 1).

## Results and Discussion

### Synthesis and characterization of NCs and DTX-Ag-NCPs

**Figure 2:**
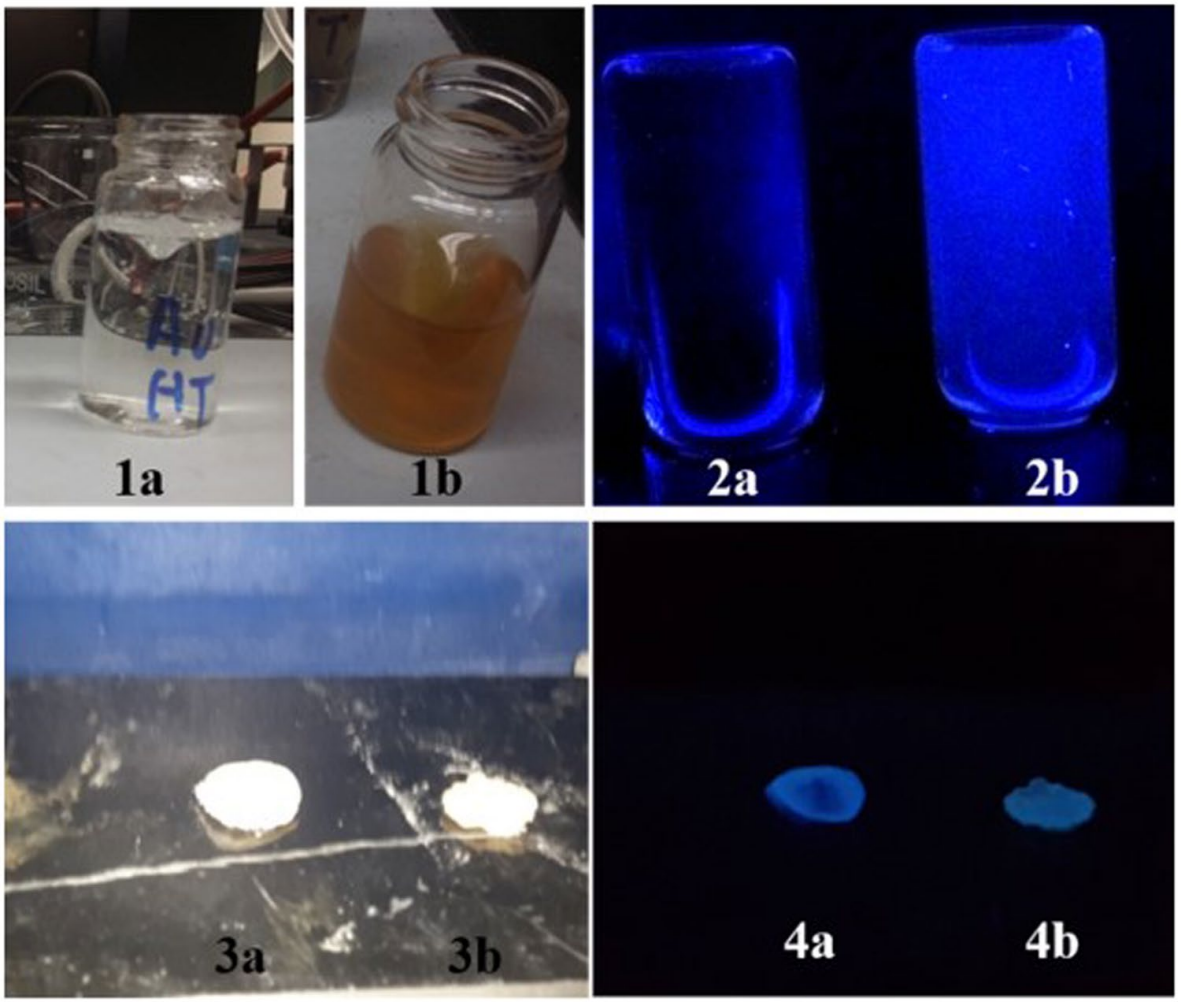
Synthesis of silver nanoclesters (NCs) and DTX-Ag-NCPs = Docetaxel loaded and NCs conjugated thilated chitosan hybrid nanoclusters (DTX-Ag-NCPs) (**1a**) before microwave treatment, (**1b**) after microwave treatment followed by dialysis resulting in formation of NCs, (**2a**) under UV light before synthesis, (**2b**) NCs formation with blue fluorescence, (**3a** & **3b**) lyophilized NCs & DTX-Ag-NCPs, respectively, under normal light and (**4a** & **4b**) lyophilized NCs and DTX-Ag-NCPs under UV light, respectively.

Folate grafted-thiolated CS (FA-TCS) stabilized Ag NCs with blue fluorescence were synthesized *via* microwave assisted method. The DTX-Ag-NCPs containing both the DTX and NCs were further prepared *via* ionic gelation method. Ag was able to be reduced inside the thiol pockets of branched CS polymer which was subsequently used for the DTX-Ag-NCPs preparation (Fig. 1). The NCs retained their fluorescence in solution and lyophilized state as shown in Fig. 2. The particle size, PDI and zeta potential of the NCs are shown in the Table 1. The observed hydrodynamic diameter of the DTX-Ag-NCPs was 190 nm. Furthermore, the DTX-Ag-NCPs showed homogeneity in synthesis with low polydispersity having (PDI < 0.15) and a positive zeta potential due to stabilization with cationic polymer. This positive charge could facilitate intestinal uptake of the DTX-Ag-NCPs because of anionic nature of mucous layer^22^. Moreover, the amount of elemental Ag in the DTX-Ag-NCPs was determined to be 16.58 µg/g using inductively coupled plasma mass spectrometry (ICP-MS).

**Table 1:** Physicochemical characterization of formulations synthesized showing particle size, poly dispersity, zeta potential and encapsulation efficiency.

Formulation	Particle size (nm)	Polydispersity Index (PDI)	Zeta potential (mV)	Encapsulation Efficiency (%)
NCs	42.50 ± 3.61	0.21 ± 0.15	+ 3.10 ± 1.84	−
Ag-NCPs	112.48 ± 5.87	0.18 ± 0.17	+ 18.43 ± 3.22	−
DTX-Ag-NCPs	190.72 ± 2.19	0.13 ± 0.12	+ 22.70 ± 2.22	73.65 ± 6.5

The results are shown as mean ± S.D of triplicate experiment.

Abbreviations: NCs = silver nanoclusters, Ag-NCPs = blank nanocapsule containing NCs but no drug, DTX-Ag-NCPs = Docetaxel loaded and NCs conjugated thilated chitosan hybrid nanoclusters.

### Differential scanning calorimetry (DSC), Fourier transform Infrared (FTIR) spectroscopy and X-ray diffraction (XRD) analysis

**Figure 3:**
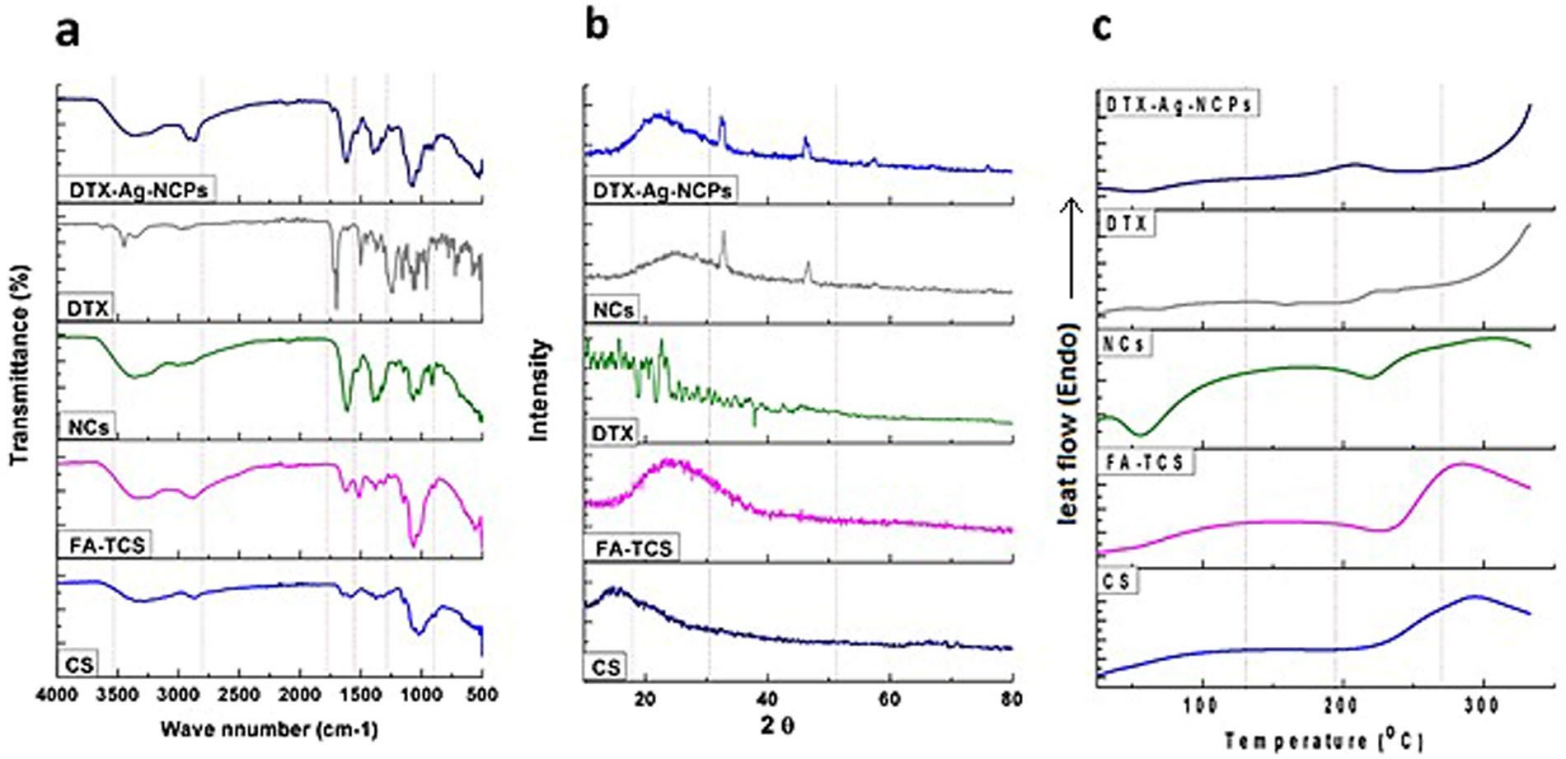
FTIR, DSC and XRD analysis (**a**) FTIR spectra showing characteristic peaks for all formulations, (**b**) XRD analysis of all the formulations representing specific peaks (**c**) DSC thermogram showing temperature effect on all formulations.

The appearance of characteristic peaks at 1656, 1590, and 1256 cm^−1^ in FTIR spectra (Fig. 3a) of CS exhibited the presence of amide I, amide II and amide III respectively. The carbonyl and thiol peaks were present respectively at 1715 cm^−1^ and 2550–2620 cm^−1^ in TGA and absent in CS as reveled in our previous study. The carbonyl peaks were observed to disappear in TCS, which was a derivative of TGA and CS and this clearly indicates the bond formation between –COOH groups of TGA and -OH or -NH groups of CS. Likewise in FA-TCS, which is a derivative of TCS and FA, all the peaks with some shift in position and low in intensity pointed out the chemical bonding between carboxylic groups of folic acid and amino moieties of TCS. The –(SH) peaks were finally present in FA-TCS at 2595 cm^−1^, which afterwards were observed to diminish in IR spectrum of FA-TCS protected AgNCs. The covalent linkages of silver or gold with thiol (-SH) groups are well-known and thus it is assumed that the FA-TCS having terminating thiol functionalities is responsible for clusters formation. The presence of NCs in the DTX-Ag-NCPs resulted a significant shift in the stretching peaks of amide bands at 1656 and 1590 cm^−1 ^^23^. The chemical integrity of DTX was confirmed by the characteristic stretching peaks appearing in FTIR spectra (Fig. 3a) at 3449, 3351 and 1713 cm^−1^, indicating the presence of functionalities like N-H, O-H and C=O respectively. The comparison of FTIR spectrum of DTX with that of NCs and the DTX-Ag-NCPs revealed the presence of chemically unmodified DTX in DTX-Ag-NCPs^19^.

Furthermore, the XRD pattern in (Fig. 3b) showed characteristic reflection of NCs at 34° and 44°, which slightly diminished in the DTX-Ag-NCPs in the presence of DTX. The characteristic XRD patterns of pure DTX disappearing in XRD spectra of the DTX-Ag-NCPs indicated its presence in amorphous state^24^.

The DSC analysis was performed to check any change in physical state of pure DTX and encapsulated NCs. The DTX showed melting point around 169 °C (Fig. 3c), which was not observed in the DTX-Ag-NCPs suggesting the presence of DTX in amorphous form within NCs which is supportive towards improved aqueous solubility and oral bioavailability of hydrophobic crystalline drug^25^.

### Scanning electron microscope (SEM) and energy dispersive X-ray (EDX) Analysis

**Figure 4:**
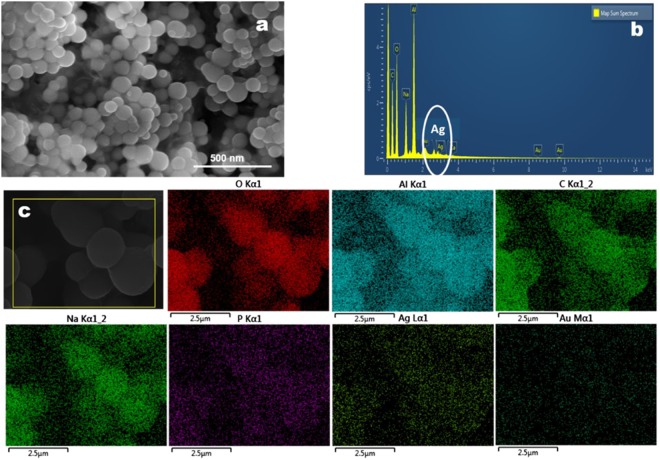
SEM/EDX analysis of DTX-Ag-NCPs (**a**) SEM images of DTX-Ag-NCPs, (**b**) spot EDX spectra of DTX-Ag-NCPs showing Ag and other metals in terms of percentage, (**c**) EDX analysis of DTX-Ag-NCPs showing different element within DTX-Ag-NCPs.

SEM and EDX analysis revealed spherical morphology of the DTX-Ag-NCPs with particle size of around 175 nm in diameter (Fig. 4a). The EDX spectra of the DTX-Ag-NCPs (Fig. 4b) shows the elemental peaks and signal of Ag inside the DTX-Ag-NCPs which confirmed the incorporation of Ag in the form of NCs inside the capsules. This was also confirmed with the elemental peaks observed point and ID scan of its EDX analysis as shown in Fig. 4c and Table S1.

### Optical Behavior

**Figure 5:**
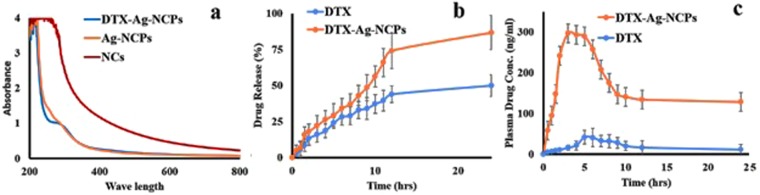
(**a**) UV-absorbance spectra of NCs, Ag-NCPs and DTX-Ag-NCPs showing no plasmonic response with NCs and Ag-NCPs whereas, slight deviation and appearance of curve in case of DTX-Ag-NCPs (**b**) *In vitro* drug release studies showing cumulative percentage drug release from DTX-Ag-NCPs and DTX suspension in 2 M phosphate buffer at 37 °C against time over period of 24 h and (**c**) Oral bioavailability study of DTX suspension and DTX-Ag-NCPs in rabbit (n = 5) showing the plasma drug concentration after oral administration of 10 mg/kg of formulations and blood withdrawn at predefined time interval was analyzed through HPLC. Error bar represents Mean ± S.D. of three experiments.

The nanoclusters do not show any plasmonic properties as reported due to their extremely small size and molecule like behavior having discontinuous and discrete energy levels^26^. However, slight deviation i.e. appearance of a new peak was observed in DTX-Ag-NCPs which corresponds to DTX. Therefore, there was no effect of DTX on Ag-NCs inside the DTX-Ag-NCPs (Fig. 5a).

### Encapsulation Efficiency

The encapsulation efficiency is an important factor to be determined for a good formulation to be developed. The encapsulation efficiency of DTX in the DTX-Ag-NCPs was observed to be 73.65% which was considered to be very good for a hydrophobic drug^27^.

### *In vitro* DTX Release

Once successfully encapsulated, drug must come out of the DTX-Ag-NCPs to produce effect at target site. In the current study, the *in vitro* DTX release from the DTX-Ag-NCPs was studied for 24 h (Fig. 5b). DTX being hydrophobic in nature, was available about 53% from pure DTX suspension. Whereas, a sustained and consistent release of DTX (>80%) from the DTX-Ag-NCPs was calculated for 24 h. This release pattern of DTX was because of the gradual swelling of FA-TCS which increases solubility of DTX leading to a sustained release to maintain plasma level over a longer period of time.

To further probe into the release mechanism, different mathematical models were applied to the dissolution data. The results, based upon R^2^ values are shown in Table S2 and revealed the release mechanism from the DTX-Ag-NCPs followed Korsmeyer-Peppas model and the value of release exponent (*n* = 0.67) suggested the release mechanism as anomalous transport involving erosion and diffusion from polymer^28^.

### Cytotoxicity Synergism

The NCs were investigated for the cytotoxic synergism with DTX. Epithelial tumors of lungs, breast and colon have shown overexpressed folate receptors, thus, presenting potential tumor targeting site by folate conjugated nanoparticles^29^. Once attached to the folate receptor, the carrier-receptor complex is internalized and initiates the effect. The cytotoxic effect of these DTX-Ag-NCPs was assessed against human breast cancer (MDA-MB-231) cell line.

**Figure 6:**
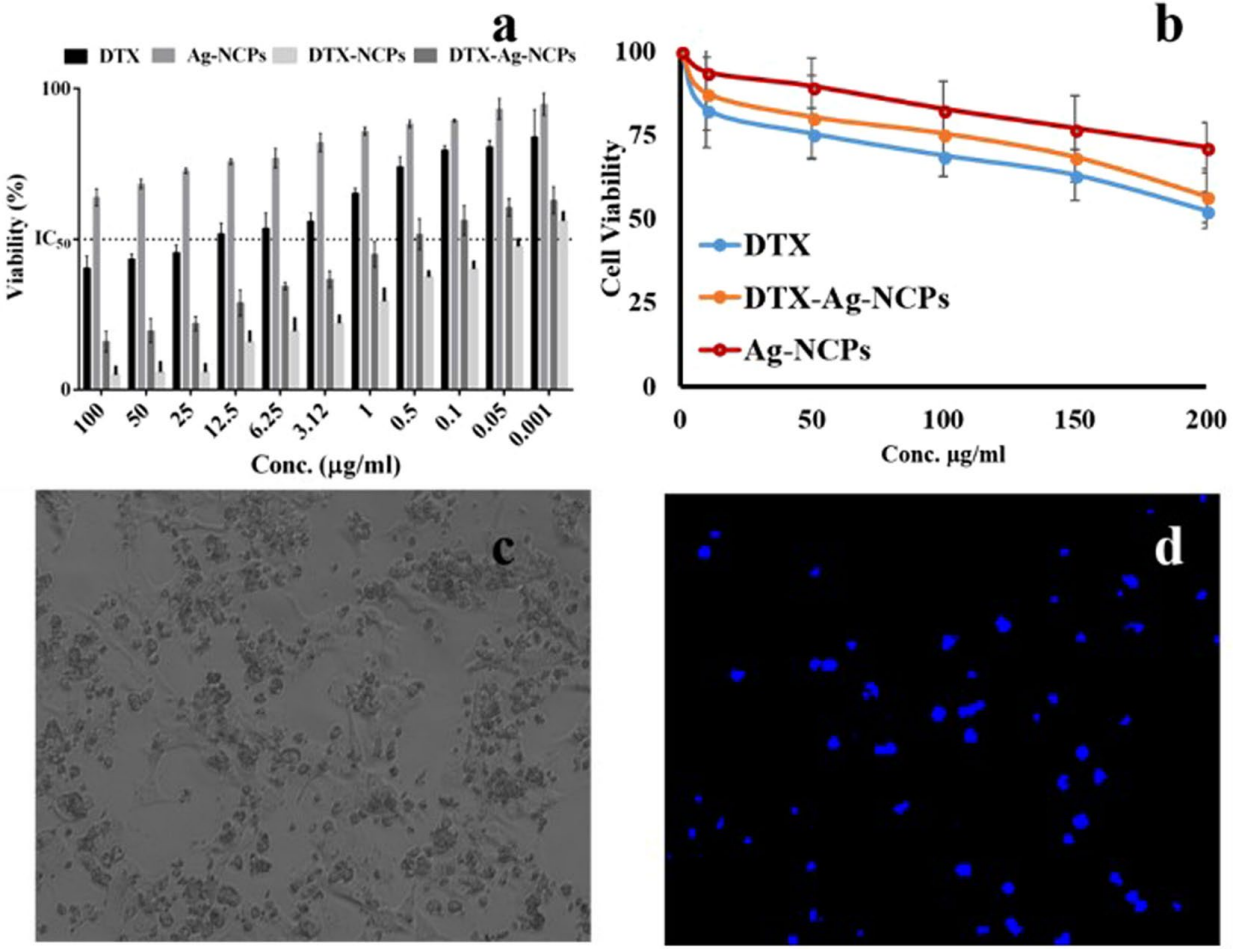
*In vitro* cytotoxicity and imaging studies against (**a**) human breast cancer cell line (MDA-MB-231) and (**b**) human macrophages isolated from fresh human blood, using different concentrations of DTX suspension, DTX-Ag-NCPs and Ag-NCPs to check anti-cancer activity and biocompatibility respectively (**c**) bright field cellular image and (**d**) under UV-light showing fluorescence and cell death after 24 h MTT assay. Error bar represents Mean ± S.D. of three experiments.

DTX suspension showed (Fig. 6a) anti-cancer activity against MDA-MB 231 with IC_50_ value of 14.32 µg/ml because of hydrophobic nature which retarded its release and permeation into the cells. Ag-NCPs, without drug, also exerted cytotoxicity to cells although lower than the DTX and its IC_50_ value was beyond 100 µg/ml. Interestingly, the DTX-Ag-NCPs containing both Ag and DTX were found to be the most effective with the lowest IC_50_ value of 0.0427 µg/ml as compared to NCs showing higher anti-cancer potential at lower concentrations making it more potent. This manifold lower IC_50_ value might be due to synergistic cytotoxic effect of DTX and Ag containing NCs within the DTX-Ag-NCPs. Also, the presence of thiol groups on their surface may also have resulted in their improved internalization through efflux pumps inhibition^30^. The DTX-NCPs showed lower anticancer activity (0.536 µg/ml) as compared to DTX-Ag-NCPs (0.062 µg/ml) suggesting their superiority as final formulation with synergistic anti-cancer activity.

### Escape from immune system

Biocompatibility assessment is an important parameter to study body’s response towards the formulation once it is inside the body either for shorter or longer durations. Silver is known to have toxic potential and could lead to severe damage to liver, kidney, lungs or spleen depending upon their exposed concentration^31^. These toxic effects could be minimized or avoided by surface modification of these metal-based formulations or capping them with some biocompatible moieties. The biocompatibility of the formulation was checked *in vitro* using fresh human macrophages. Anti-cancer drug loaded nanoparticles are primarily captured by macrophages which make them a suitable candidate for evaluation of the immune response^32^. The results showed concentration dependent cytotoxicity of all the treatment (Fig. 6b). Ag- NCPs showed more than 80% viability at 50 µg/ml as compared to around 65% viability with DTX at the same concentration. However, the toxicity of Ag-NCPs was increased at higher concentrations, which may be attributed to their increased internalization as compared to pure DTX. Ag-NCPs showed around 70% viability even at higher concentrations. The slight higher biocompatibility of, the DTX-Ag-NCPs, is to human cells might attribute to the fact of being present in polymeric scaffold.

### Oral Bioavailability

Oral bioavailability and pharmacokinetics were studied in healthy rabbits of either sex. Ideally, nanoparticles should be around 300 nm in size to reach systemic circulation *via* different intestinal mechanism^33^. The DTX-Ag-NCPs were, therefore, aimed to increase the solubility and permeability of hydrophobic DTX in rabbits. From the plasma level-concentration data (Fig. 5c), pharmacokinetic parameters were calculated (Table 2). It was observed that after oral administration, DTX suspension reached C_max_ after 5 h and remained above the minimum effective concentration (35 ng/mL) for 3 h only. On the other hand, the DTX-Ag-NCPs attained the minimum effective concentration (MEC, 35 ng/ml) levels within 3 h and remained within therapeutic window for 24 h due to sustained release profile. The maximum level was achieved after 3 hours. It was also observed that DTX level from the DTX-Ag-NCPs was many folds below the minimum toxic concentration (2700 ng/mL) as observed after intravenous administration of DTX reported in literature^34^. Plasma half-life (t_1/2_) of NCs was 123.5 h, which was around 6.8 folds higher than that of pure DTX i.e. 18.03. This prolonged half-life in plasma may be due to stronger mucoadhesion of thiol modified CS based nanoparticles DTX-Ag-NCOs^34^. C_max_ was also increased 6 folds as compared to that with pure DTX^35–37^. The AUC_0-24_ showed high increase in oral bioavailability i.e. 8.89-folds as compared to pure DTX. This enhanced oral bioavailability of DTX from the DTX-Ag-NCPs might be due to combination of factors such as increased mucoadhesion, enhanced retention time in gut, enhanced paracellular transport because of thiolated polymer on the surface of nanocarriers and the sustained release from DTX-Ag-NCPs. The particle size around 300 nm might have also facilitated the paracellular transport through gastric mucosa^38^.

**Table 2:** Different pharmacokinetic parameters calculated from plasma level-time curve obtained after oral administration of DTX suspension and NCs to rabbits.

Pk Parameter	Unit	DTX	DTX-Ag-NCPs
Tmax	h	5	3
Cmax	ng/ml	41.78	297.39
t1/2	h	18.039	123.46
AUC_0–24_	ng/ml*h	440.70	3921.46
AUMC_0-inf_obs_	ng/ml*h^2	19100.51	4664644.30
MRT_0-inf_obs_	h	26.16	174.08
Cl/_F_obs_	(mg)/(ng/ml)/h	0.014	0.00037
F		1	8.89

Abbreviations: DTX-Ag-NCPs = Docetaxel loaded and NCs conjugated thilated chitosan hybrid nanoclusters, DTX = Docetaxel solution (control), Tmax = time to reach maximum concentration, Cmax = maximum concentration in blood, t_1/2_ = half life in blood, AUC_0–24_ = area under the curve from time 0 to 24 h, *AUMC*_0-inf_obs_ = , MRT_0-inf_obs_ = mean residence time observed, C1/F_obs_ = , F = fraction.

### Acute Oral Toxicity Evaluation

Acute oral toxicity of the formulations was performed on Swiss albino mice^39^. DTX, DTX-Ag-NCPs and Ag-NCPs (10 mg/kg each) were administered orally to each mice. Mice were observed for 48 h after administration and no visible changes in skin, fur or behavioral pattern were observed. Afterwards, same parameters were observed every day to determine any toxic event macroscopically. No mortality and significant change in body weights was observed for 14 days. After 14 days, the blood was withdrawn from each mice and pooled for CBC and serum biochemistry analysis^40^.

### Serum Biochemistry Analysis

**Figure 7:**
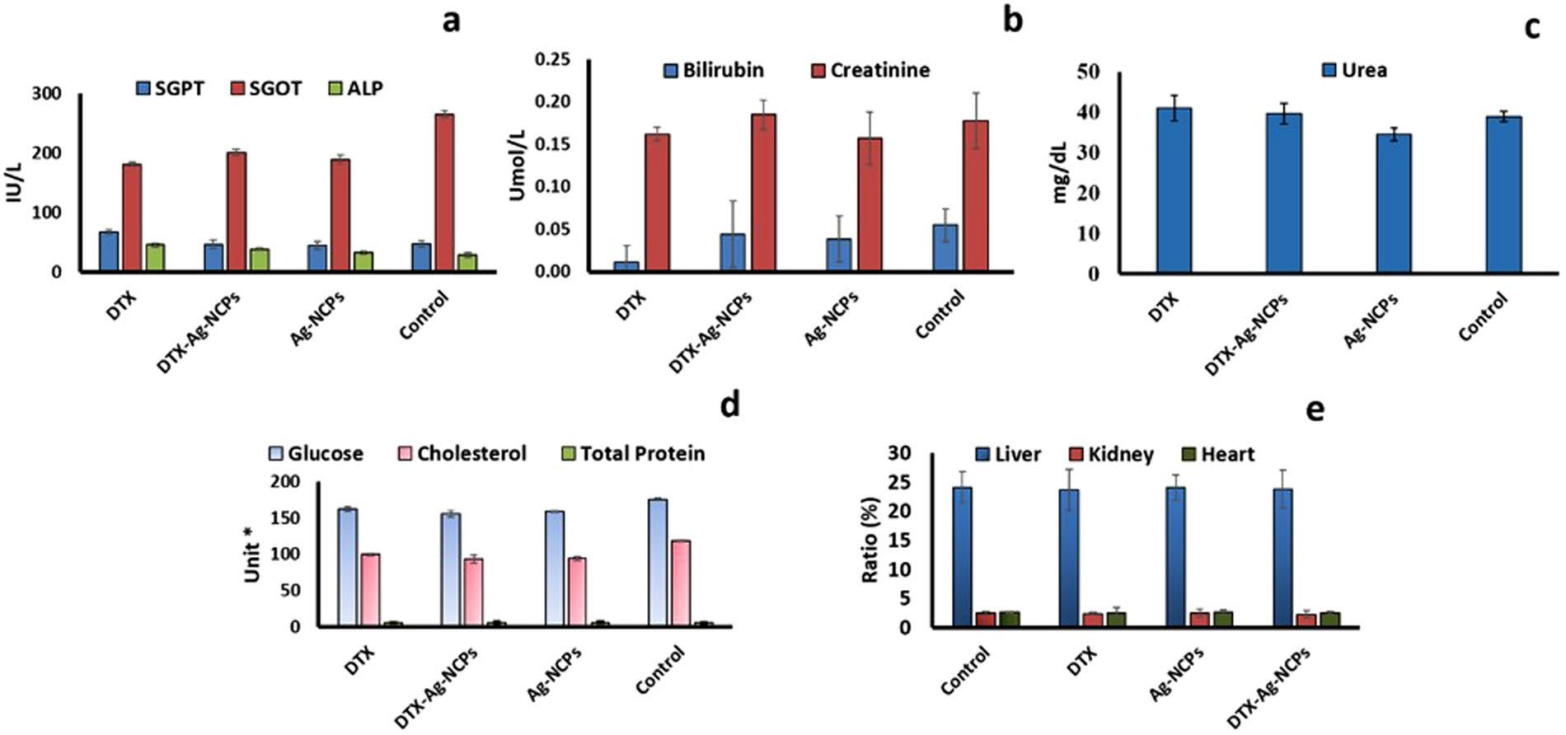
Serum biochemistry of mice blood determining acute oral toxicity (**a**) Liver function tests, (**b**) bilirubin and creatinine, (**c**) urea, (**d**) serum biochemistry and (d) organ to body weight analysis performed on Swiss albino mice, after DTX, DTX-Ag-NCPs and Ag-NCPs in accordance with OECD 425 guidelines for acute oral toxicity. Error bar represents Mean ± S.D. of three experiment. (*units for: Glucose mg/dL, Cholesterol mg/dL, Total protein g/dL).

Serum biochemistry was performed on the plasma obtained after centrifugation. The LFTs results in Fig. 7b showed no significant changes in bilirubin level compared to control. The level was observed slightly higher for the DTX-Ag-NCPs as compared to Ag-NCPs and DTX, but fell within the acceptable limits^3^. Serum glutamic pyruvic transaminase (SGPT) was observed at the highest level in DTX treatment as compared to that with Ag-NCPs and NCs treatment (Fig. 7a). Serum glutamic oxaloacetic transaminase (SGOT) level was increased with all treatments and the maximum level was found with the DTX-Ag-NCPs among the three treatments which might be due to the detoxification of foreign particles (Fig. 6a). Alkaline phosphatase (ALP) level (Fig. 7a) was decreased from 45 to 38 IU/L with drug encapsulated in DTX-Ag-NCPs. Except for the SGOT and ALP, the other parameters did not produce significant changes in serum level indicating the safe use of NCs in the presence of NCs and DTX.

The results for RFTs showed that blood urea nitrogen (BUN) level was slightly increased (Fig. 7c) with the DTX and the DTX-Ag-NCPs but remained within the limits. However, this level was slightly decreased with Ag-NCPs as compared to that for the control. The creatinine level was slightly raised with the DTX-Ag-NCPs when compared to control, yet, was found within limits. Contrary to that, a slight decrease in creatinine level with DTX and Ag-NCPs was observed but fell within the acceptable limits. The effect of the formulation was also investigated on serum glucose, cholesterol and total protein level (Fig. 7d). The DTX-Ag-NCPs significantly decreased the serum glucose and cholesterol level as compared to the other two treatment groups, which also decreased the serum glucose and cholesterol level. Total protein remained unaffected with all the treatments. The effect of formulation was evaluated on blood and its components through complete blood count (CBC). The result (Table S3) showed that the effect on different blood components was markedly decreased by the DTX-Ag-NCPs as compared to pure DTX, which has more toxic potential against RBCs and platelets. The Ag-NCPs blank did not produce any significant effect on the CBC and remained closer to the results for control group.

### Organ to Weight Ratio

Organ weights are regarded as a good parameter of *in vivo* toxicity studies. Any change in organ to body ratio is correlated to the treatment associated effects^41^. The results (Fig. 7e) showed no significant changes for any of the three organs i.e. liver, kidney and heart. However, there was a minute change observed within liver only with the DTX-Ag-NCPs which was not significant as compared to control. Over all, there was no significant effect on organs as observed by the treatment during acute oral toxicity testing.

### Histopathological Evaluation

**Figure 8:**
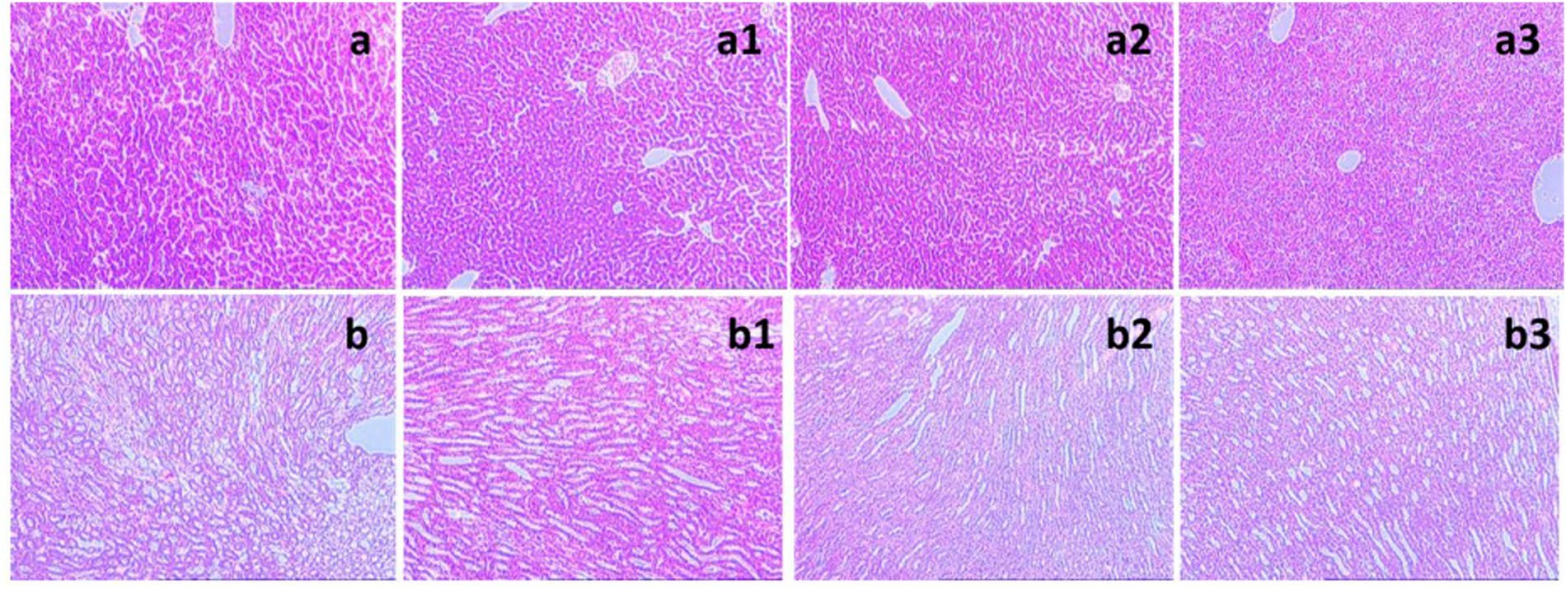
Microscopic evaluation of tissue histology; (**a**) Control liver, (a1) treatment with DTX, (a2) treatment with DTX-Ag-NCPs (a3) treatment with Ag-NCPs and (**b**) Control kidney, (b1) treatment with DTX, (b2) treatment with DTX-Ag-NCPs, (b3) treatment with Ag-NCPs obtained from Swiss albino mice after being euthanize.

The macroscopic examination of liver and kidney did not show any visible changes or lesions on these organs. To explore further, histological investigations were performed on the slides prepared from liver (Fig. 8a-a3) and kidney (Fig. 8b-b3). The microscopic examination of the slides did not show any changes like necrosis or fatty changes in the liver, and similarly, kidney appeared to be normal as compared to control. Thus, the DTX-Ag-NCPs did not produce any toxicity on liver and kidney and support the results obtained from LFTs and RFTs indicating the safety of these DTX-Ag-NCPs.

### Stability Studies

The poor long term stability due to fluctuations in temperature and humidity may affect physicochemical properties of drug and nano formulations^42^. Generally, stability is ensured by freezing or lyophilizing the nano formulation in the presence of cryoprotectant that prevents aggregation or structural deformation of nanoparticles upon long term storage^43^. The stability of nanoparticles has been evaluated using different conditions following ICH guidelines Q1A (R2) and lyophilized state by different research groups out of which 4 °C and 37 °C have been reported most suitable for polymeric nanoparticles^44^. NCs and the DTX-Ag-NCPs were examined for their stability over a period of 3 months while refrigerated at 4 °C under dark condition. The stability was checked in terms of any changes in their particle size, zeta potential and PDI. The results shown in table S4 present the stability data of particle size, zeta potential and PDI measured for both formulations for 3 months. The particle size was changed slightly which might be because of aggregation of the DTX-Ag-NCPs that also resulted in slight change in PDI after 3 months. No significant change in zeta potential was observed. The data showed high stability of the formulation at low temperature as compared to high temperature.

## Materials and Methods

### Materials

All chemicals used in the research were purchased from Sigma-Aldrich, Germany, and Docetaxel (DTX) was received as gift from NovaMed Pharmaceuticals Pvt. Ltd. All the solvents used were of analytical and HPLC grade.

### Methods

The details on synthesis of FA-TCS, particle size, zeta potential, DSC, XRD, FTIR, optical evaluation, *in vitro* release, biocompatibility, oral bioavailability and stability is provided in supplementary material.

All the experiments including animals were performed in accordance with *OECD* 425 and institutional ethical guidelines. All protocols were approved from Bio-Ethical Committee of Quaid-i-Azam University Islamabad, Pakistan (Protocol No. BEC-FBS-QAU-20 and BEC-FBS-QAU-21).

### Synthesis of silver nanoclusters (NCs)

50 mg of FA-TCS and 20 mg EDTA were dissolved in 5 ml of deionized water. silver nitrate (0.5 mM, 1 mL) was added to this solution and stirred for 15 min. Afterwards, the reaction mixture was irradiated with microwaves for 2 min operating at 100 W using microwave reactor (CEM; Discoverer, USA). The resulting solution was then purified using dialysis membrane (2,000 MWCO) for 24 h with deionized water exchanged at regular intervals of 6 h. The yellowish brown colloidal dispersion obtained at the end indicated the formation of silver nanoclusters (NCs). The solution was afterwards stored under dark conditions in refrigerator until further used^45^.

### Preparation of hybrid nanocapsules DTX-Ag-NCPs

Hybrid nanocapsules (DTX-Ag-NCPs) containing both DTX and NCs were synthesized through ionic gelation technique^46^. DTX (1 mg) was added to solution of NCs (5 ml) under continuous stirring followed by addition 0.1% solution of Tween-80 to increase wettability of DTX. After 15 min, cross linker sodium tripolyphosphate (TPP) solution (1%) was added drop wise to above mixture to synthesize DTX-Ag-NCPs. The solution was stirred for further 4 h followed by dialysis to remove any unreacted materials.

Same method was used to synthesize blank nanocapsules (Ag-NCPs) without DTX to serve as control in different experiments. The Ag-NCPs and the DTX-Ag-NCPs were stored in refrigerator under dark conditions till further use.

### SEM/EDX Analysis

Surface morphology and elemental analysis of the DTX-Ag-NCPs was studied through scanning electron microscope (FEI Nova NanoSEM 450, Thermo Fisher, USA) equipped with EDX operating between 15–25 kV with working distance of 5 mm. The samples for SEM/EDX analysis were prepared on carbon coated copper grid followed by blotting a drop of 1% ammonium molybdate solution. For better contrast, the dried sample was further coated with gold, using sputter coater (Desk V HP, Denton, USA, operating at 40 mA for 15 sec under vacuum. Afterwards, the sample was analyzed for SEM and EDX results^47^.

### Cytotoxic Synergism Studies

*In vitro* cytotoxicity synergistic potential of the DTX-Ag-NCPs was explored and compared with NCs and control DTX suspension through MTT assay using breast cancer (MDA-MB-231) cell line^11,22^. The cells were seeded in 96-well plate having 6000 cells/well in fetal bovine serum supplemented Dulbecco’s Modified Eagles Medium (DMEM/FBS) solution. The cells were incubated with different concentrations of formulations of DTX, NCs, and the DTX-Ag-NCPs containing equivalent amount of DTX and Ag-NCPs as internal control for 24 h. Untreated cells with 100% viability were taken as control and the cells without addition of MTT were used as blank to calibrate the instrument. IC_50_ values for each formulation was calculated using Graphpad Prism 6.02 software^19^.

### Acute Oral Toxicity

Female Swiss albino mice weighing 32 ± 5 g, were divided into 4 groups (n = 5) and were kept to free access of food and water at controlled environment. The group 1 was given DTX suspension, group 2 was given the DTX-Ag-NCPs and group 3 was given Ag-NCPs, whereas group 4 was given N/S and served as control. The dose (10 mg/kg) was administered orally through gavage. The mice were kept under observation for 48 h for change in weight and visual observations for mortality, behavior pattern (fur and skin, consistency of feces, urination color, salivation, eyes, respiration, sleep pattern, mucous membrane, convulsions, and coma), physical appearance changes and sign of illness on every day of the study period^48^. After 14 days, the blood was withdrawn for serum biochemistry analysis and mice were sacrificed for tissue histology studies^49^.

### Plasma Biochemistry and Hematology Analysis

After 14 days, the complete blood count (CBC) was performed on blood and plasma was carefully collected and LFTs, RFTs, glucose, total protein and cholesterol were analyzed^50^.

### Organ to Body Ratio

After 14 days, the mice were sacrificed and the vital organs (heart, kidneys and liver) were removed, carefully washed with normal saline and individually weighed. These weights were compared with the organ’s weight from the control group to calculate the organ-body weight index using the formula below:^48,51^

$$Organ\,body\,weight\,index=100\times \frac{\mathrm{Organ}\,\mathrm{weight}\,}{\mathrm{Body}\,\mathrm{weight}}$$

### Histopathology of Vital Organs

The previously removed and washed organs were macroscopically examined for any abnormalities/lesions against control. Later, the organs were fixed in paraffin blocks and sections (0.5 µm) were cut carefully using rotary microtome and fixed on glass slide, stained and microscopically (Olympus BX51M) examined for any evident sign of toxicity.

### Statistical Analysis

All the experiments were done in triplicates to reduce the chances of error and establish significant correlation between the data obtained. Data are presented as Mean ± SD using SPSS 21 and Graphpad Prism 6.1. The *p* value less than 0.05 (*p < 0.05) was considered to indicate the significant difference^22^.

## Conclusion

In this study, hybrid nanocapsules (DTX-Ag-NCPs) were synthesized using folate grafted thiolated CS for the co-delivery of Ag NCs and DTX. The synthesized DTX-Ag-NCPs offered enhanced oral drug delivery potential of DTX (sustained blood level for 24 h, 6 folds higher half-life and 9 folds higher bioavailability) as compared to DTX suspension and cytotoxic synergism against MDA-MB-231 breast cancer cell line. These improved properties of the DTX-Ag-NCPs can be attributed to co-delivery of low quantity of NCs and DTX in the presence of thiolated CS, to potentiate the pharmacological effects (~300 folds low IC_50_). The acute oral toxicity studies of these formulations containing 16.58 µg/g of elemental silver, following oral administration, showed no significant effects on different parameters i.e., CBC and serum biochemistry analysis and histological evaluation of major organs. Therefore, all the studied parameters suggested that the DTX-Ag-NCPs have great potential to serve as a versatile and efficient carrier for oral delivery of Docetaxel with increased activity against breast cancer.

## Electronic supplementary material

Supplementary information
