# Supplementary material for: Polymeric nanocapsules embedded with ultra-small silver nanoclusters for synergistic pharmacology and improved oral delivery of Docetaxel
Source: Sci Rep. 2018 Sep 6;8:13304. doi: 10.1038/s41598-018-30749-3 (PMC6127092; doi:10.1038/s41598-018-30749-3)
Supplement: Supplementary file 1 — Supplementary information [file 41598_2018_30749_MOESM1_ESM.docx]

**Supplementary Information**

**Polymeric nanocapsules embedded with ultra-small silver nanoclusters for synergistic pharmacology and improved oral delivery of Docetaxel**

**Authors**

Muhammad Farhan Sohail^a,b,c^, Syed Zajif Hussain^b^, Hamid Saeed^d^, Ibrahim Javed^b,e^, Hafiz Shoaib Sarwar^a,f^, Akhtar Nadhman^g^, Zil-e-Huma^b^, Mubashar Rehman^h,^, Sarwat Jahan^j^, Irshad Hussain^b,i*^, Gul Shahnaz^a*^

^a^ Department of Pharmacy, Faculty of Biological Sciences, Quaid-i-Azam University, Islamabad, 45320, Pakistan.

^b^ Department of Chemistry and Chemical Engineering, SBA School of Science and Engineering (SBASSE), Lahore University of Management Sciences (LUMS), Lahore, 54792, Pakistan.

^c^ Riphah Institute of Pharmaceutical Sciences, Riphah International University, Lahore Campus, Lahore, Pakistan

^d^ College of Pharmacy, University of the Punjab, Allama Iqbal Campus, Lahore, Pakistan.

^e^ ARC Centre of Excellence in Convergent Bio-Nano Science and Technology, Monash Institute of Pharmaceutical Sciences, Monash University, 381 Royal Parade, Parkville, VIC 3052, Australia

^f^ Department of Pathology, Ohio State University Medical Center, Columbus, OH, USA

^g^ Institute of Integrative Biosciences, CECOS University, Phase VI, Hayatabad, Peshawar, Pakistan.

^h^ Department of Pharmacy, University of Lahore – Gujrat Campus, Gujrat 50700, Pakistan

^i^ US-Pakistan Center for Advanced Studies in Energy (USPCAS-E), University of Engineering & Technology (UET), Peshawar, Pakistan.

^j^ Department of Animal Sciences, Faculty of Biological Sciences, Quaid-i-Azam University, Islamabad, 45320, Pakistan.

***Corresponding Authors**

Gul Shahnaz

E-mail Address: [gshahnaz@qau.edu.pk](mailto:gshahnaz@qau.edu.pk)

Irshad Hussain

E-mail Address: [ihussain@lums.edu.pk](mailto:ihussain@lums.edu.pk)

**Experimental**

**Materials**

All chemicals including silver nitrate, sodium hydroxide, sodium borohydride, low molecular weight chitosan, thioglycolic acid (TGA) 99%, sodium tripolyphosphate (TPP), 5,5-dithiobis(2-nitrobenzoic acid), 1-ethyl-3-(3-dimethylaminopropyl)carbodiimide (EDAC), disodium di hydrogen phosphate, sodium dihydrogen phosphate, glucose, sodium chloride, potassium chloride, sodium borohydride, magnesium chloride, trehalose, fetal bovine serum (FBS), Dulbecco’s modified Eagle medium (DMEM) and 3-(4,5-Dimethylthiazolyl-2)-2,5-diphnyltetrazolium bromide (MTT) were purchased from Sigma-Aldrich (Germany). Dialysis membranes having cutoff values 2,000 and 12,000 MWCO were also purchased from Sigma-Aldrich (Germany). Docetaxel (DTX) was received as gift from NovaMed Pharmaceuticals Pvt. Ltd., Pakistan. All the solvents used were of analytical and HPLC grade.

**Methods**

*Synthesis of Folate Grafted Thiolated Chitosan*

Folate grafted thiolated chitosan (FA-TCS) was synthesized by following the method previously reported by us ^1^. Briefly, thiolated chitosan was synthesized by dissolving chitosan (1.0 g) in acetic acid solution (100 ml, 0.1%) followed by addition of TGA (1.0 g) with continuous stirring until completely dissolved. Thereafter, 1-ethyl-3-(3-dimethylaminopropyl) carbodiimide (50mM) was added with subsequent addition of hydroxylamine (50 mM) to prevent oxidation during synthesis. The pH of resulted reaction mixture was adjusted to 5.0 using HCl (2 M) and kept under stirring for 4 hours at 25^º^C ± 2 to complete the reaction. To eliminate the unreacted material and purify the TCS, the mixture was dialyzed 5 times in dark at 10ºC using dialysis membrane with cut off value 12 KDa. Lastly, the TCS solution was freeze dried using lyophilizer (Scanvac; coolsafe 110, laabogen, Denmark) and stored at 4^o^C. In the next step, folic acid was grafted on TCS by dissolving the 1 g TCS in 100 ml deionized water followed by addition of folic acid and EDAC solution in DMSO. The pH of the reaction mixture was adjusted to 9 with NaOH (0.5 M) and stirred for 16 h. Afterwards, FA-TCS was purified through dialysis as described previously using phosphate buffer (pH=7.0), lyophilized and stored at 4°C until further use.

*Particle Size and Zeta Potential Measurement*

Hydrodynamic radius and zeta potential of silver nanoclusters (NCs), Docetaxel-NCs conjugated thiolated chitosan hybrid nanocapsules (DTX-Ag-NCPs) and blank NC-thiolated chitosan hybrid nanocapsules (Ag-NCPs) were measured by dynamic light scattering measurements using Zetasizer (Malvern, NanoZSP, UK).

*DSC, FTIR and XRD Analysis*

Differential Scanning Calorimetry (DSC) analysis was performed to check the stability and possible interactions of DTX and all other ingredients of DTX-Ag-NCPs in temperature range of 20─350^o^C, at a rate of 10ºC/min under air purge of 10 ml/min, using DSC (TA Instruments, SD Q600). The stability of drug was also studied by Fourier Transformed Infra-Red (FTIR) spectroscopy using FTIR spectrophotometer (Bruker, alph-P, USA). The amorphous nature of DTX and presence of Ag in DTX-Ag-NCPs were confirmed by XRD patterns obtained in angle range of 20–70° through Powder X-ray diffractometer (Bruker, D2 Phaser, USA) operated at; 30kV, 10mA, and using Cu *Kα1* radiations (λ = 1.5406 Å) with the step scan size of 0.0505°.

*Optical Evaluation*

Absorption spectra of NCs and DTX-Ag-NCPs was analyzed using multiplate reader (Perkin-Elmer, EnSpire Multimode Plate Reader) and UV visible spectrophotometer (Shimadzu, UV-1800, Japan).

*Encapsulation Efficiency*

The quantity of encapsulated DTX in DTX-Ag-NCPs was calculated using method reported earlier through HPLC analysis ^1,2^. For this, all formulations were analyzed using RP-HPLC (waters), with isocratic mobile phase having flow rate of 0.8 ml/min at 230 nm, by repeating each in triplicate and average was calculated to determine encapsulation efficiency from all formulations by using the formula:

$$Encapsulation efficiency \left( \% \right)=\frac{amount of drug in formulation}{total amount of drug}x 100$$

*In Vitro Drug Release Studies*

*In-vitro* release of DTX from DTX-Ag-NCPs was studied through dialysis membrane diffusion technique. The weighed quantity of formulations containing drug equivalent to 2 mg was re-suspended in deionized water and placed in dialysis bag (Cutoff value 12kDa), sealed and immersed in 25 ml of phosphate buffer (0.1 M; pH 2.0) containing tween 80 (1%; w/v) at 37±0.5^o^C under stirring at 100 rpm. DTX suspension was used as standard to compare the drug release from formulations. Samples were collected at predefined intervals, filtered through syringe filter having pore size of 0.22 µm and analyzed through HPLC (waters e2695) using the same method ^3,4^ The release kinetics was studied through DDSolver, a free Microsoft Excel Add-in.^5^

*Biocompatibility*

The biocompatibility of the NCs and DTX-Ag-NCPs was assessed against the fresh human macrophages. The macrophages were isolated from fresh human blood using Ficoll-Gastrografin gradient method.^6^ Macrophages were suspended in RPMI media and incubated in CO_2_ incubator. Viable macrophages were seeded in 96-well plate and treated with different concentrations of NCs, DTX-Ag-NCPs and Ag-NCPs to access the biocompatibility. After 24h incubation, trypan blue assay was performed to count the viable cells and IC_50_ was calculated for each formulation.

*Oral Bioavailability*

All the animal studies were conducted in compliance to the approved protocol of Bio-Ethical Committee of Quaid-i-Azam University Islamabad, Pakistan (Protocol No. BEC-FBS-QAU-20). Oral bioavailability studies of NCs were conducted in rabbits. The rabbits were divided into 3 groups (n=5) and kept in the animal house with free access to food and water. Group 1 was given DTX-Ag-NCPs, group 2 was given DTX suspension and group 3 was given N/S to serve as a control. The samples (10mg/kg) were orally administered through gavage needle. Blood samples were withdrawn from ear marginal vein of each rabbit at predefined time interval using 1 ml sterile syringe each time. The plasma was separated from blood samples and stored at -20 ºC till further used for analysis.^7,8^ The drug was extracted from plasma samples and was analyzed using HPLC method described earlier.

*Stability Studies*

Stability of NCs and DTX-Ag-NCPs was analyzed for change in physical appearance by examining the change in particle size, PDI and zeta potential over a period of 3 months while keeping them refrigerated at 4 ºC under dark conditions.^9^

**Result and Discussion**

*Synthesis and characterization of FA-TCS*

Thiolated chitosan has been reported to possess several advantages including increased mucoadhesion, permeation enhancement thorough para cellular routes and P-gp inhibition as compared to chitosan.^1,10^ Folate receptors are found on all cell membranes, however they are over expressed in many of the tumor cell surface.^11^ These properties of thiol and folic acid were combined to achieve folate receptor targeting through oral route. Therefore, folate grafted thiolated chitosan was successfully synthesized by EDAC coupling method in a two-step process i.e. synthesis of thiolated chitosan (TCS) and subsequent folate grafting (FA-TCS). EDAC activated the carboxylic group of thioglycolic acid and folic acid which were conjugated to amine of chitosan backbone through amide linkage resulting in organic scaffold for further experiments.^12,13^ The dried FA-TCS appeared as yellowish fibrous material and was stored at 4°C.

**Table S1:** EDX analysis showing percentage of various elements detected in DTX-Ag-NCPs.

| **Element** | **Line Type** | **Apparent Concentration** | **k Ratio** | **Wt%** | **Wt% Sigma** | **Standard Label** | **Factory Standard** |
| --- | --- | --- | --- | --- | --- | --- | --- |
| C | K series | 2.93 | 0.02935 | 16.96 | 21.08 | C Vit | Yes |
| O | K series | 42.37 | 0.14259 | 44.92 | 11.41 | SiO2 | Yes |
| Na | K series | 9.89 | 0.04176 | 9.81 | 2.5 | Albite | Yes |
| Mg | K series | 0.46 | 0.00308 | 0.62 | 0.17 | MgO | Yes |
| Al | K series | 16.29 | 0.117 | 19.58 | 5 | Al2O3 | Yes |
| P | K series | 2.42 | 0.01356 | 2.12 | 0.54 | GaP | Yes |
| Cl | K series | 0.84 | 0.00735 | 0.02 | 0.26 | NaCl | Yes |
| K | K series | 0.24 | 0.002 | 0.26 | 0.08 | KBr | Yes |
| Ca | K series | 0.16 | 0.00143 | 0.18 | 0.06 | Wollastonite | Yes |
| Pd | L series | 1.09 | 0.01092 | 1.47 | 0.43 | Pd | Yes |
| Ag | L series | 0.18 | 0.00162 | 2.23 | 0.05 | Ag | Yes |
| Au | M series | 1.85 | 0.01846 | 1.83 | 0.76 | Au | Yes |
| Total: |  |  |  | 100 |  |  |  |

**Table S2:** Pharmacokinetic modeling showing different mathematical models applied on dissolution data to determine the mechanism of release from DTX-Ag-NCPs

| **Formulation** | **Zero Order** | | **Korsmeyer-Peppas** | | **Higuchi** | | **Hixon-Crowell** | |
| --- | --- | --- | --- | --- | --- | --- | --- | --- |
|  | R^2^ | K_o_ | R^2^ | N | R^2^ | K_H_ | R^2^ | K_HC_ |
| DTX | 0.67 | 3.06 | 0.95 | 0.56 | 0.95 | 10.98 | 0.85 | 0.01 |
| DTX-Ag-NCPs | 0.82 | 4.80 | 0.98 | 0.67 | 0.91 | 16.72 | 0.96 | 0.02 |

**Table S3:** Complete blood count (CBC) analysis of mice blood obtained after 14 days’ acute oral toxicity analysis. The results from blood of 5 mice are shown as mean ± SD.

| **Paramaeter** | **DTX** | **DTX-Ag-NCPs** | **Ag-NCPs** | **Control** |
| --- | --- | --- | --- | --- |
| **RBC** | 6.86 ± 4.91 | 8.01 ± 5.67 | 7.83 ± 4.15 | 8.22 ± 4.92 |
| **MCV** | 57.57 ± 5.52 | 55.46 ± 5.36 | 54.59 ± 8.32 | 56.84 ± 2.28 |
| **MCH** | 15.54 ± 4.35 | 15.44 ± 7.41 | 18.69 ± 6.24 | 16.56 ± 4.61 |
| **PCV** | 54.37 ± 11.93 | 73.88 ± 8.49 | 78.17 ± 12.63 | 50.92 ± 6.82 |
| **Hb** | 13.20 ± 6.37 | 14.97 ± 2.18 | 14.19 ± 5.25 | 15.44 ± 5.14 |
| **WBC** | 12.45 ± 7.63 | 13.73 ± 7.07 | 13.69 ± 6.08 | 14.28 ± 7.37 |
| **Platelets** | 632 ± 87.57 | 707 ± 78.54 | 677.33 ± 64.04 | 723.66 ± 89.23 |
| **RDW %** | 16.92 ± 6.63 | 16.69 ± 5.31 | 17.46 ± 5.29 | 17.05 ± 6.70 |
| **MPV** | 7.11 ± 4.91 | 6.63 ± 6.43 | 7.49 ± 4.33 | 6.76 ± 7.17 |

**Table S4:** 3-month stability studies data showing changes in particle size and PDI of Ag-NCPs and DTX-Ag-NCPs stored in dark at 4 °C.

| **Formulation** | **Temp (°C)** | **Particle size**  **(nm)** | | | **Polydispersity Index**  **(PDI)** | | | **Zeta Potential**  **(meV)** | | |
| --- | --- | --- | --- | --- | --- | --- | --- | --- | --- | --- |
|  |  | **1 month** | **2 month** | **3 month** | **1 month** | **2 month** | **3 month** | **1 month** | **2 month** | **3 month** |
| Ag-NCPs | 4 | 112.48 ± 5.87 | 123.50 ± 4.84 | 128.30 ± 4.72 | 0.18 ± 0.17^*^ | 0.20 ± 0.18 | 0.24 ± 0.15 | 17.13± 4.17 | 17.20 ± 2.45 | 15.36 ± 2.76 |
| DTX-Ag-NCPs |  | 190.72 ± 2.19 | 196.40 ± 3.20 | 211.60 ± 4.75 | 0.13 ± 0.12^*^ | 0.16 ± 0.14 | 0.17 ± 0.19^*^ | 21.88 ± 3.42 | 20.71 ± 4.38 | 18.40 ± 3.56 |

**References**

1 Sohail, M. F. *et al.* Folate grafted thiolated chitosan enveloped nanoliposomes with enhanced oral bioavailability and anticancer activity of docetaxel. *Journal of Materials Chemistry B* **4**, 6240-6248, doi:10.1039/C6TB01348A (2016).

2 Saboktakin, M. R., Tabatabaie, R. M., Maharramov, A. & Ramazanov, M. A. Development and in vitro evaluation of thiolated chitosan—poly (methacrylic acid) nanoparticles as a local mucoadhesive delivery system. *International journal of biological macromolecules* **48**, 403-407 (2011).

3 Lee, S. J. *et al.* Tumor-targeting glycol chitosan nanoparticles as a platform delivery carrier in cancer diagnosis and therapy. *Nanomedicine* **9**, 1697-1713 (2014).

4 Saremi, S., Atyabi, F., Akhlaghi, S. P., Ostad, S. N. & Dinarvand, R. Thiolated chitosan nanoparticles for enhancing oral absorption of docetaxel: preparation, in vitro and ex vivo evaluation. *Int J Nanomedicine* **6**, 119-128 (2011).

5 Muhammad Farhan Sohail *et al.* Formulation and Development of Flurbiprofen Microencapsules by Modified Solvent Evaporation Technique and its In-Vitro Evaluation. *Trop J Pharm Res* **13**, 1031-1038 (2014).

6 Khan, H. *et al.* In‐vitro antileishmanial potential of peptide drug hirudin. *Chemical biology & drug design* **89**, 67-73 (2017).

7 Venkatesh, D. N. *et al.* Fabrication and in vivo evaluation of Nelfinavir loaded PLGA nanoparticles for enhancing oral bioavailability and therapeutic effect. *Saudi Pharmaceutical Journal* (2015).

8 Jiao, Y. *et al.* In vitro and in vivo evaluation of oral heparin–loaded polymeric nanoparticles in rabbits. *Circulation* **105**, 230-235 (2002).

9 Jain, A., Thakur, K., Kush, P. & Jain, U. K. Docetaxel loaded chitosan nanoparticles: Formulation, characterization and cytotoxicity studies. *International journal of biological macromolecules* **69**, 546-553 (2014).

10 Bonengel, S. & Bernkop-Schnürch, A. Thiomers—From bench to market. *Journal of Controlled Release* **195**, 120-129 (2014).

11 Lu, Y. & Low, P. S. Folate-mediated delivery of macromolecular anticancer therapeutic agents. *Advanced drug delivery reviews* **64**, 342-352 (2012).

12 Wan, A., Sun, Y. & Li, H. Characterization of folate-graft-chitosan as a scaffold for nitric oxide release. *International journal of biological macromolecules* **43**, 415-421 (2008).

13 Iqbal, J. *et al.* Thiolated chitosan: development and in vivo evaluation of an oral delivery system for leuprolide. *European Journal of Pharmaceutics and Biopharmaceutics* **80**, 95-102 (2012).
